# Multitasking Rab Proteins in Autophagy and Membrane Trafficking: A Focus on Rab33b

**DOI:** 10.3390/ijms20163916

**Published:** 2019-08-12

**Authors:** Niamh E. Morgan, Meritxell B. Cutrona, Jeremy C. Simpson

**Affiliations:** School of Biology and Environmental Science & Conway Institute of Biomolecular and Biomedical Research, University College Dublin (UCD), D04 N2E5 Dublin, Ireland

**Keywords:** membrane traffic, autophagy, Rab GTPase, Rab33b

## Abstract

Autophagy (particularly macroautophagy) is a bulk degradation process used by eukaryotic cells in order to maintain adequate energy levels and cellular homeostasis through the delivery of long-lived proteins and organelles to the lysosome, resulting in their degradation. It is becoming increasingly clear that many of the molecular requirements to fulfil autophagy intersect with those of conventional and unconventional membrane trafficking pathways. Of particular interest is the dependence of these processes on multiple members of the Rab family of small GTP binding proteins. Rab33b is a protein that localises to the Golgi apparatus and has suggested functions in both membrane trafficking and autophagic processes. Interestingly, mutations in the *RAB33B* gene have been reported to cause the severe skeletal disorder, Smith–McCort Dysplasia; however, the molecular basis for Rab33b in this disorder remains to be determined. In this review, we focus on the current knowledge of the participation of Rab33b and its interacting partners in membrane trafficking and macroautophagy, and speculate on how its function, and dysfunction, may contribute to human disease.

## 1. Rab Proteins as Molecular Coordinators of Membrane Trafficking

Eukaryotic cells have evolved an extensive internal membrane apparatus that comprises the endoplasmic reticulum (ER), the Golgi apparatus and various classes of endosomes and lysosomes. This endomembrane system needs to support communication between individual compartments, ensuring the coordinated flow of material to the appropriate destinations. Broadly, these membrane trafficking pathways regulate protein and lipid cargo movement inwards and outwards of the cell. The biosynthetic, or secretory pathway, is considered to be the conventional route of protein secretion [1], and is responsible for the manufacturing, processing and shipment of cargo that is synthesised in the ER towards destinations including other compartments of the endomembrane system itself, as well as the plasma membrane (PM) and the extracellular space. This membrane flow is countered by the endocytic pathway, which coordinates the uptake and/or internalisation of molecules from the PM. In parallel to these pathways, discoveries over the last two decades have established that an increasing number of proteins bypass the conventional secretory pathway using an unconventional transport route, independent of the ER and Golgi apparatus [2,3,4]. 

Among the different classes of molecular machinery that regulate membrane trafficking throughout the endomembrane system, the small soluble Ras-related proteins in brain (Rab) serve key functions [5,6]. The Rabs comprise the largest family of small GTP binding proteins within the Ras superfamily, with over 60 members identified in humans [7,8]. Within the context of membrane traffic they assist in a variety of steps, including budding, movement, docking, tethering and fusion of transport carriers operating between the various endomembrane compartments of the cell [9]. This assortment of function is possible thanks to the timely recruitment of specific effector proteins such as cargo adaptors, motor proteins, tethers and fusion-driving machinery at various stages of membrane traffic events. The recruitment of effectors relies on the GTP/GDP binding state of the Rab (i.e., ‘active’ and ‘inactive’ status), hence Rabs can be considered as membrane-associated molecular switches. In this GTPase cycle, GDP to GTP exchange is catalysed by guanine nucleotide exchange factors (GEFs), which cause a conformational change and the exposure of a lipid tail that allows anchorage of the Rab to a membrane. Once in the GTP-bound form, Rabs can recruit or activate multiple effector molecules. Conversion of the Rab back to its GDP-bound form occurs through GTP hydrolysis, driven by both the intrinsic GTPase activity within the Rab protein and also enhanced by GTPase-activating proteins (GAPs). At steady state, Rabs are predominantly found loaded with GDP, and are bound to a protein termed the Rab GDP dissociation inhibitor (GDI). Targeting of the Rab-GDI complex to specific membranes is also thought to involve a family of transmembrane proteins known as GDI displacement factors (GDFs). Since Rab proteins can rapidly associate and dissociate with membranes through this guanine nucleotide exchange and hydrolysis mechanism, their distribution, and in turn their functionality, is highly dynamic. As such, the specific cellular localisation patterns of Rab proteins are key in establishing organelle identity [10,11]. 

It is now appreciated that Rab effectors play a fundamental role in enabling Rabs to function in events beyond their classical role in membrane transport. In particular, recent studies show the functional involvement of many members of this GTPase family in pathways such as autophagy [12], as discussed below.

## 2. Brief Overview of Autophagosome Formation

Macroautophagy, the major type of autophagy, is a bulk degradation process used by eukaryotic cells for maintenance of its organelles, managing energy levels and cellular homeostasis. This pathway involves the formation of a transient double-membrane structure, called the phagophore or isolation membrane (IM), which acts as a compartment to actively sequester material for degradation. Following expansion and closure, this structure becomes an autophagosome, eventually fusing with the lysosome/vacuole, leading to the degradation of the engulfed material completing the degradative process [13]. The ‘core’ autophagy-related genes (ATGs) represent the fundamental machinery for the biogenesis of autophagosomes and are conserved across eukaryotic cells. Detailed reviews of the autophagic process and the molecular action of ATGs have been described extensively elsewhere [14,15,16]. 

The proteins encoded by the ATGs assemble into functional complexes, and the initiation of autophagy is orchestrated by their activation and recruitment to specific membranes [14,17]. Autophagy can be induced in response to changes in the extracellular environment, classically by nutrient depletion [18], as well as other stimuli, including intracellular DNA damage and ER stress [19,20,21,22]. The target of rapamycin complex 1 (TORC1) plays a primary role in one of the signalling pathways that senses changes in nutrient availability [23]. Nutrient starvation initiates an intracellular signalling cascade by discontinuing TORC1 stimulation, resulting in the activation of the Atg1 kinase complex in yeast, and a similar serine-threonine kinase complex in mammalian cells; the ULK1 complex (ULK1, ULK2, ATG13, RBCC1/FIP200 (RB1-inducible coiled-coil protein 1), ATG101) [24]. These events trigger the initiation of IM formation. The ULK1 complex translocates to autophagy initiation sites that might originate from an initial cup-shaped membrane subdomain of the ER called the omegasome; which is enriched in phosphatidylinositol 3-phosphate (PI(3)P) [25], and regulates the recruitment of a second complex for nucleation of the IM, namely the class III phosphatidylinositol 3-kinase complex 1 (PI3KC3-C1). PI3KC3-C1 consists of a catalytic subunit, the lipid kinase vacuolar protein sorting 34 (VPS34), and regulatory subunits Atg14L, VPS15 and beclin 1 (Atg6 in yeast). IM elongation and autophagosome completion requires two ubiquitin-like conjugation pathways. In the first pathway, sequential reactions by the E1 enzyme Atg7 and the E2 enzyme Atg10 conjugate Atg12 to the lysine residue in Atg5. Atg16L, a WD-repeat-containing molecule that is an essential component of the autophagic pathway, interacts non-covalently with Atg5 resulting in a ternary complex which is essential for the formation of pre-autophagosomes. The second pathway drives conjugation of members of the Atg8 family (i.e., microtubule-associated protein 1 light chain 3 (MAP-LC3; referred to as LC3 hereafter)) LC3-I (cytoplasmic) to the lipid phosphatidylethanolamine (PE), forming LC3-II, which localises to isolation membranes and autophagosomes through a process mediated by Atg7 and Atg3 [26]. Additionally the Atg16L/12/5 complex acts as an E3-ubiquitin ligase that enhances LC3 conjugation to PE [27,28]. Lipidation of LC3 governs the incorporation of LC3-II into the IM, required for expansion and closure of the pre-autophagosomal membrane, which eventually results in cargo recruitment and formation of the autophagosome. Atg9 is a transmembrane member of the core ATGs, and is required for autophagosome formation and enlargement, and in turn is a key regulator of autophagy induction, however its precise role is unclear. The interactions highlighted above are vital steps that underlie autophagosome assembly. Although many questions about autophagosome initiation and formation remain, studies suggest that membranes derived from the ER, mitochondria, ER-mitochondria contact sites, Golgi apparatus, endocytic vesicles, recycling endosomes and the plasma membrane may all contribute to the autophagosome (reviewed in [29]).

## 3. Regulation of Autophagy by Rab Proteins

**Figure 1:**
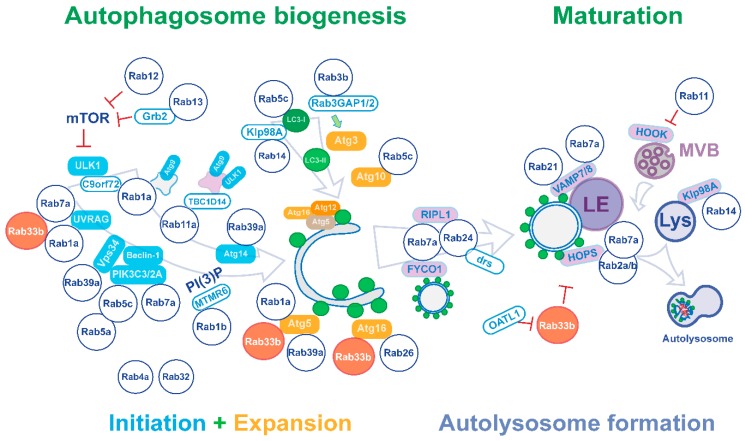
Regulation of the autophagy pathway by Rab proteins. Sequential steps of autophagy (from left to right) involve members of the Rab GTPase protein family that regulate this pathway through their effectors. Biogenesis of autophagosomes is driven by autophagy proteins that promote initiation of isolation membrane (IM) formation (light blue) and subsequently sustain expansion of IM structures (orange). Maturation steps occur following transport and ultimate fusion of mature autophagosomes with membranes of the endo-lysosomal system (light violet) including multivesicular bodies (MVBs), late endosomes (LEs) and mature lysosomes (Lys). The involvement of Rab proteins at different stages of the autophagy pathway is represented as direct interaction of a given Rab with proteins that control the autophagy pathway (solid colour rectangles), indirect interaction with autophagy proteins through a Rab effector (white rectangles) or as a means of functional regulation (inhibition or stimulation arrows). Rab33b (indicated in red) contributes to multiple steps of the autophagy pathway through distinct effectors (i.e., UVRAG, Atg5, Atg16L, OATL1), which supports IM initiation and expansion at early stages, and is eventually involved in later stages of autophagosome maturation via modulation of the fusion of autophagosomes with lysosomes.

A significant body of evidence has shown that the autophagic process is largely achieved by a series of dynamic membrane trafficking events, using many components associated with membrane trafficking. The action of several Rab proteins has been shown to regulate autophagosome formation, elongation, transport and ultimate fusion with lysosomes, all of which are essential to canonical ‘degradative’ autophagy [17] (Figure 1). From several studies in mammalian cells, including a proteomic analysis aimed to catalogue the autophagy interaction landscape [30], various autophagy-associated proteins have been found to bind to a number of Rabs, indicating that ATGs constitute direct Rab effectors. A list of the Rab GTPases found in mammals that have been identified to contribute to both early and late stages of autophagy are shown in Table 1, with those Rabs functioning in multiple stages of the autophagic process highlighted in grey. Rab proteins, many with Atg proteins as interacting partners, have been found to orchestrate essential processes involved in early autophagic steps for IM elongation and autophagosome formation; Rabs have been shown to modulate TORC1 or Vps34/Beclin1-PIK3C3 activation; assist in the translocation of ULK1 complex- and Atg9-containing vesicles; and promote Atg5-Atg12 conjugation and recruitment of Atg16L or LC3 into pre-autophagosomal membranes, all of which are essential elements of autophagosome formation.

At later stages of autophagy, autophagosomes mature through fusion with multi-vesicular bodies (MVB) to generate amphisomes. Amphisomes receive membrane inputs from both early and late endosomes, and eventually fuse with lysosomes for maturation into autolysosomes. This process is assumed to proceed in a similar, if not identical manner to that of endocytic fusion events, and the molecules and their functions that confer lysosomal membrane identity are similarly required for both endosome- and autophagosome-lysosome fusion [66]. Since Rab GTPase effectors include molecular motors and membrane fusion machinery, it is no surprise that Rabs participating in endocytic transport (i.e., Rab5, Rab7, Rab11, Rab14 and Rab21) are also involved in autophagosome maturation and sustaining the autophagic flux (see Table 1 and Figure 1). Interestingly, Rabs that function in retrograde transport from the Golgi to the ER, namely Rab2 and Rab33b, also play a role in fusion of autophagosomes with late endosomes and lysosomes [65,67]. Rab8a and Rab25 are also involved in autophagy, but their roles are less clear [12].

One Rab family member, Rab33b, is particularly attractive as a multitasking regulator in multiple autophagy and membrane trafficking events. Although it has been shown to be necessary for both these cellular processes, its specific role is not yet defined. One reason for interest in Rab33b specifically, is that its dysfunction has been linked to a rare skeletal disease, Smith–McCort Dysplasia (SMC), therefore highlighting important unsolved questions around the complete functional role of Rab33b. Here we also summarise what is currently known regarding the mechanisms by which Rab33b may operate in trafficking and autophagic processes.

## 4. Membrane Trafficking Roles of Rab33b

In mammals, there are two members of the Rab33 subfamily; Rab33a and Rab33b. The *RAB33B* gene is universally expressed [68], whereas *RAB33A* expression is restricted to neuronal cells, lymphocytes and melanocytes only [69,70,71], but in both cases the genes are organised in a similar fashion from two exons [68,69]. Sequencing data acquired by Zheng and colleagues showed Rab33a and Rab33b proteins share unique amino acid sequences at their effector binding domain, and sequence conservation downstream of the G-3 region extending to the α-helix 3/loop 7 region, previously shown to be conserved among specific Rab subclasses. Rab33 subclass-specific characteristics have been conserved through evolution; exemplified by homologues in *Caenorhabditis elegans* and *Ciona intestinalis* [69]. Taken together, these two genes represent their own subclass within the Rab family, however given that the overall amino acid identity between Rab33a and Rab33b is low at 55.3% and that their expression patterns differ, it is likely that they have divergent functions. Rab33a has been shown to function in membrane trafficking events in neurons, where it localises to the Golgi apparatus and mediates anterograde trafficking of post-Golgi carriers for membrane exocytosis and axon outgrowth, as well as vesicle exocytosis in other cells lines [72,73,74]. Rab33b localises to the medial Golgi cisternae and has previously been demonstrated to function at the Golgi apparatus [68,75,76]. 

Retrograde trafficking from the Golgi apparatus to the ER is essential to recover ER-resident proteins that have escaped from the ER during the first steps in the process of secretion [77]. Such ER proteins are recognised by defined motifs called retrieval signals, which in turn bind to receptors and associated transport machinery, to ensure their rapid return to the ER. The primary cellular machinery driving this event is a cytosolic complex of proteins together termed the coat protein complex I (COPI), which acts as a physical driver of membrane reorganisation and cargo selection at the level of the Golgi. However, Golgi-to-ER transport can also occur in a COPI-independent manner that is dependent on another Rab GTPase, Rab6a, and it is this mechanism that certain Golgi-resident enzymes such as glycosyltransferases, and in addition opportunistic protein toxins such as the Shiga and Shiga-like toxins, use to reach the ER [78]. 

**Figure 2:**
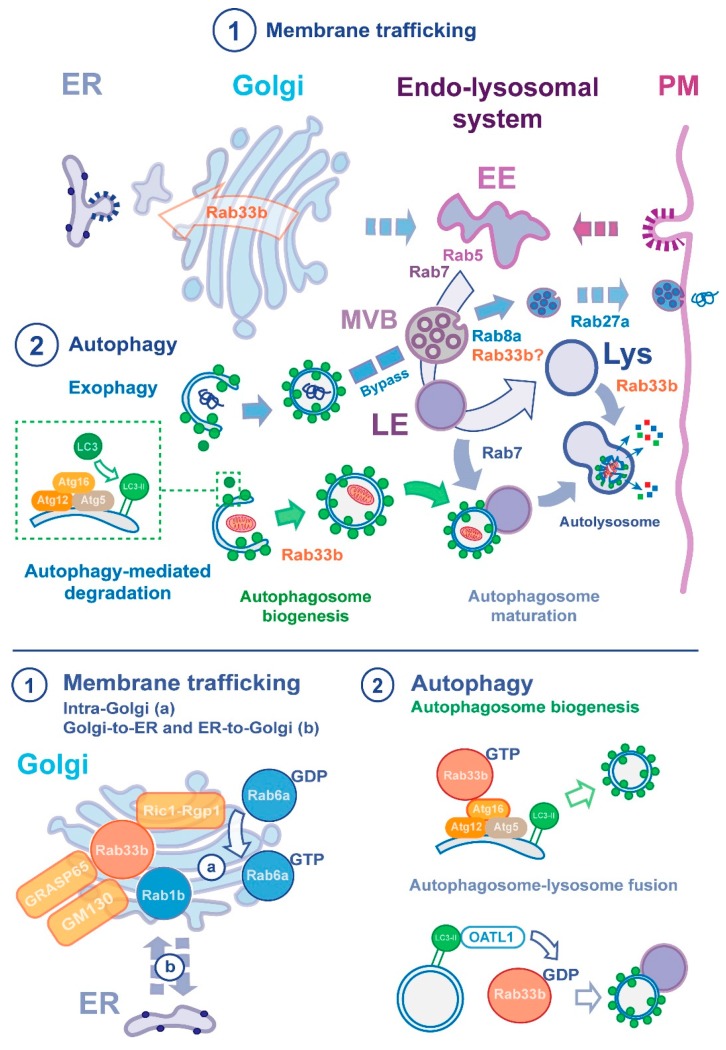
Rab33b at the intersection of membrane trafficking and autophagy. Key vesicle trafficking and autophagy pathways (upper panel). Compartmentalisation and establishment of stations along the endo-lysosomal system occur through a maturation process from early endosomes (EEs), multivesicular bodies (MVBs) and late endosomes (LEs) to mature lysosomes (Lys). A Rab cascade, that involves recruitment of Rab7 to Rab5 domains, leads to formation of a stable LE compartment before delivery to lysosomes. Autophagy-mediated degradation converges with this pathway. Biogenesis of autophagosomes at dedicated foci, culminates with recruitment of lipidated LC3 (green boxed inset) to pre-autophagosomal membranes. Next, autophagosome maturation results from the transport of autophagosomes towards EEs and LEs, and fusogenic events at distinct stages of the endosomal maturation pathway. Rabs participating in endocytic transport (i.e., Rab7) sustain the autophagic flux. Degradation of cytosolic components engulfed by autophagosomes occurs by fusion of autophagosomes or amphisomes that result from the fusion between autophagosomes and LEs with lysosomes. The resulting autolysosomes mediate the breakdown of autophagic cargo. A secondary arm of the autophagic pathway, also known as exophagy, plays a role in unconventional protein secretion. Rab proteins provide a regulatory role that avoids stages that result in autolysosomal formation for autophagosomes carrying unconventional secretory cargo. Autophagosomal secretion relies on fusion of autophagosomes with MVBs and subsequent fusion of these intermediates with the plasma membrane (PM). Blue arrows, biosynthetic transport; purple arrows, fusiogenic autolysosomal maturation steps. Rab33b, initially described to reside in the Golgi apparatus, contributes to multiple pathways through distinct effectors. Specific roles in vesicular trafficking and autophagy are indicated in the figure (lower panel, left and right side respectively). Rab33b localises to the medial Golgi cisternae and works alongside Rab6a and has an established role in (a) the structural organisation of the Golgi apparatus, and (b) retrograde Golgi-to-ER transport. Rab33b is the first Rab protein reported to interact with Atg16L for regulation of autophagosome formation. The function of a regulator of Rab33b, OATL, suggests a functional involvement of this GTPase in later stages of autophagy-mediated degradation.

Rab33b has been shown to function in Golgi-to-ER retrograde trafficking in a similar manner to Rab6a (illustrated in Figure 2). Overexpression of either wild-type (WT) or the activated Rab33b Q92L mutant causes enhanced redistribution of Golgi-resident enzyme *N*-acetylgalactosaminyltransferase-2 (Gal*N*Ac-T2) back to the ER [76]. Conversely, overexpression of the inactivated Rab33b T47N mutant inhibits Golgi-to-ER transport of *E. coli* Shiga-like toxin subunit B (SLTxB) and does not cause a redistribution of Gal*N*Ac-T2 from the Golgi to the ER [76]. Depletion of Rab33b by RNA interference (RNAi) also suppressed intra-Golgi transport of SLTxB from *trans*- to *cis*-Golgi [75,76,79]. Taken together these results establish a functional role for Rab33b at the Golgi apparatus.

Rab33b has been functionally implicated in the organisation of the Golgi ribbon and intra-Golgi transport in conjunction with Rab6a (Figure 2). Depletion of Rab33b by RNAi, or overexpression of inactivated Rab33b, suppressed Golgi fragmentation caused by loss of ZW10 and COG3 (retrograde tethering proteins involved in Golgi organisation) [80], establishing that Rab33b and Rab6a both contribute to ZW10- and COG3-dependent Golgi ribbon organisation [79]. Moreover, the GTP-Rab6a-induced redistribution of Golgi enzymes back to the ER is dependent on Rab33b, whereas the GTP-Rab33b-induced redistribution does not require Rab6a, indicating that Rab33b acts at a later stage to Rab6a in the retrograde trafficking pathway [79]. Other work has shed light on the functional overlap of Rab33b and Rab6a in a Rab cascade between the medial- and the *trans*-Golgi. Ric1-Rgp1 has been identified as an effector of Rab33b and a GEF for Rab6a, whereby it has been shown to bind activated Rab33b, and in addition, catalyses nucleotide exchange on the late-Golgi-localised Rab6a population [81] (Figure 2). 

Additionally, a number of well-established Golgi apparatus-resident proteins have been identified as binding partners of Rab33b (Figure 2). The Golgi matrix protein GM130 has been revealed to be a direct effector of Rab33b, initially shown by it being retained on GST-Rab33b fusion protein columns in a GTP-specific manner. Preliminary findings in this study showed GRASP65, a Golgi stacking protein, to interact with Rab33b [76]. Both GM130 and GRASP65 are essential proteins for correct Golgi ribbon formation. Rab33b has also been found to interact with other Rab GTPases [82,83], namely Rab3a that is involved in regulated exocytosis and secretion [84,85,86], and Rab1b that functions in ER-to-Golgi trafficking and autophagosome formation [87,88]. Rab33b has also been demonstrated to interact with the endosomal proteins Rabaptin-5 and Rabex-5 [76]. Although the direct functional implications of these interactions have not been teased out, these findings could place Rab33b at additional, unexplored membrane-associated events. 

Together, the above findings highlight the functional and structural significance of Rab33b at the Golgi apparatus. As well as balancing protein and lipid flow through the cell, key modifications are made to most proteins as they pass through the Golgi apparatus, such as changes to their glycosylation profile, sulfation, phosphorylation, and proteolytic cleavage. Disruption in Golgi homeostasis is therefore likely to affect its function, which in turn may more widely disrupt cellular homeostasis [89]. Studies to further elucidate the functional meaning of the described Rab33b interactions may reveal additional trafficking roles played by Rab33b. 

## 5. Involvement of Rab33b Function in Multiple Steps in the Autophagic Process

Rab33b was the first Rab protein reported to be involved in the initial process of autophagosome formation through interaction with its autophagic effector Atg16L [30,50]. As the Atg16L complex is present on initiating autophagosomes, but never mature autophagosomes, it is believed to function in regulating this initial biogenic process [90,91,92]. Through a GST protein fusion assay using 60 Rab proteins as bait, it was found that Atg5, Atg12, and Atg16L, all of which are essential for IM formation, were co-purified with GTP-bound Rab33b. Subsequent analysis found that Atg16L, but not Atg5 or Atg12, binds specifically to Rab33b, and to Rab33a with a lower affinity [50]. Additionally, this study showed by immunofluorescence analysis that the overexpression of the Rab33b binding domain of Atg16L strongly inhibits autophagosome formation. Biochemical analysis demonstrated that the overexpression of the activated Rab33b mutant induces lipidation of LC3 (i.e., increase of LC3-II), an essential process in autophagosome formation, and attenuated macroautophagy evident from an increased accumulation p62/sequestosome 1 (which is expected to be degraded upon completion of autophagy). Surprisingly however, RNAi-mediated depletion of Rab33b did not affect autophagosome formation [50]. Taken together, these findings suggest that Rab33b acts as a modulator in autophagosome formation through interaction with Atg16L; however, the mechanism of this remains to be established.

Atg16L appears to be responsible for the IM localisation of the LC3 complex [27], however the mechanism of targeting, membrane source and way in which the Atg16L complex regulates autophagosome biogenesis has not yet been elucidated. The identification of Golgi-localised Rab33b as an interactor of Atg16L, and the finding that the overexpression of the Rab33b binding domain of Atg16L localises to the Golgi, may indicate a potential mechanism [50]. It has been speculated by the authors of the above work that Rab33b-mediated vesicles from the Golgi apparatus provides a membrane source to the elongating IM, and the Rab33b-Atg16L interaction facilitates tethering and/or fusion of vesicles to the IM. 

Rab33b has also been found to function in later stages of autophagy. In the process of autophagosome maturation, ornithine aminotransferase-like 1 (OATL1, also known as TBC1D25) is recruited to the IM and autophagosomes through direct interaction with Atg8 homologues, and through its GAP activity is involved in the fusion between autophagosomes and lysosomes. OATL1 was identified as a GAP for Rab33b whereby it leads to Rab33b inactivation [65]. This work found that the overexpression of the wild-type or activated Rab33b mutant inhibited fusion between autophagosomes and lysosomes. The authors suggest that OATL1 is recruited to autophagosomes (through direct interaction with Atg8 homologues) and then inactivates Rab33b, leading to the fusion of autophagosomes with lysosomes. 

The link between Rab33b and OATL1 has been further supported by evidence that both of these proteins are required for the uptake and transfer of synthetic nanoparticles to lysosomal compartments. RNAi-induced depletion of Rab33b reduces the accumulation of nanoparticles in lysosomes, but this can be rescued by the overexpression of wild-type Rab33b protein [93]. Additionally, overexpression of OATL1 was also found to reduce nanoparticle accumulation in acidic compartments [93]. Taken together, overexpression of OATL1 reduces the delivery of nanoparticles to lysosomal compartments, as well as the fusion of autophagosomes with lysosomes, both of which are processes that require Rab33b. As the overexpression of either Rab33b or OATL1 inhibits lysosomal fusion events, it appears that a balance of Rab33b activity, determined in part by its GAP, is required for progression of these events. It should be noted however that much evidence for the role of Rab33b in membrane trafficking and autophagosome formation has been derived from overexpression data. Such experiments are not necessarily representative of the physiological scenario and so interpretation of experiments employing exogenously-expressed Rab mutants, which may influence multiple effector proteins, should be interpreted with caution. The known function of Rab33b in autophagy processes is illustrated in Figure 2.

## 6. An Alternative Role for Rab33 in Unconventional Secretion?

In contrast to the ‘degradative’ autophagy pathway discussed above, the autophagic machinery, through a shared but partially divergent pathway, may lead to secretion/expulsion of cytoplasmic constituents instead of their degradation. This autophagic secretory process has been termed ‘exophagy’ [94]. Exophagy enables unconventional protein secretion, which defines a trafficking route either for cytoplasmic proteins that lack an ER-signal peptide for import and which are incapable of entering the classical route for secretion, or for some integral membrane proteins, which, although they are endowed with a signal-peptide, travel to the cell surface in either a COPII- and Golgi-independent manner [2]. 

Various studies have pointed to a role for Rab33 family members and their effectors in regulating exophagic events in conjunction with their Atg binding partners. Rab33a, through its interaction with Atg16L, has been shown to regulate hormone secretion from neuroendocrine PC12 cells [95] and Rab33b in the secretion of the Hepatitis B virus (HBV) naked capsid from Huh-7 human hepatocellular carcinoma cells [96]. Release of the HBV naked capsid relies on Rab33b along with its autophagic Atg5/12/16L1 effectors, indicating Rab33b function is required for initial formation of autophagy-related membranous structures at early steps of the exophagy pathway. Moreover, in this study, it was also observed that depletion of Rab33b prevents HBV capsid release, due to suppression of apoptosis linked gene 2 interacting protein X (Alix)-assisted exocytosis [96]. Alix plays a pivotal role in control of MVB vesicle formation [97], thus blockade of capsid release in Rab33b-depleted cells may suggest the involvement of Rab33b in later stages of the MVB pathway that are important for exophagy.

Although Atg16L plays a well-established role in early autophagy, exophagic pathways of secretion occur independently of autophagic processes. This highlights a linkage between Rab33 and autophagic factors in not only autophagy, but in certain secretory events. As Rab33 variants have regulatory roles with their interactor Atg16L in both autophagic and secretory processes, it is plausible to speculate that Rab33 may act as a molecular switch to determine the fate of cargo as secretory (exophagy pathway) or degradative (autophagy pathway).

## 7. Disease and Future Perspectives

Mutations in *RAB33B* have been associated with a rare autosomal recessive spondylo-epi-metaphyseal disorder, SMC [98,99,100]. Patients with SMC present skeletal deformities consisting of marked short stature, barrel-shaped chest, disproportional length of the proximal limb, a combination of both outward and lateral curvature of the spine and small pelvis with slipped capital femoral epiphysis [101]. Individuals with mutations in *RAB33B* leading to SMC have normal cognitive intelligence [102]. This is intriguing because in a similar disorder to SMC, namely Dyggve–Melchior–Clausen syndrome (DMC), patients present with the same skeletal phenotype, but in addition show mental retardation and microcephaly. Patients with DMC have a loss-of-function mutation in the *DYM* gene, which encodes the Golgi protein Dymeclin [102]. Since no cerebral phenotype is observed in SMC patients, it seems likely that the presence of the second Rab33 isoform in the brain, namely Rab33a, may provide a compensatory mechanism resulting in a disease outcome lacking mental retardation. At the cellular level, expression of *RAB33B*-harbouring SMC-associated mutations (c.444T > A or c.136A > C) cause a striking decrease of Rab33b protein levels, suggesting a reduced protein stability that correlates with a swollen and fragmented appearance of the Golgi apparatus in dermal fibroblasts cultured from patients [98,99]. To date however, little has been revealed about the defective molecular mechanism at the cellular level that triggers this skeletal disorder.

Rab33b function has been identified as essential for a number of critical cellular events as discussed in this review. As Rab33b participates in Golgi structure and trafficking, autophagic processes and secretory processes, this could imply that failure or dysregulation of one or more of these events is involved in the pathogenesis of SMC. In line with the defective Golgi appearance observed in patients with mutations in *RAB33B*, it has been suggested that defects in proteins associated with trafficking could result in morphological changes to subcellular organelles, which in turn may affect organelle functionality [89,103]. Future studies investigating Golgi apparatus function using patient-derived cells could inform on whether the observed morphological changes reflect Golgi dysfunction, shedding light on a potential mechanism of disease.

It has been established that autophagy is important for normal cellular homeostasis, and unsurprisingly, both increased and decreased autophagy have been associated with human disease, including various neurodegenerative conditions, different cancer types and autoimmune disorders [30,31,102]. Irregularities in unconventional secretory processes have also been identified as a factor contributing to disease states, such as cancer and neurodegenerative disease [104]. As Rab33b and its effector Atg16L are required for autophagosome formation and maturation in the autophagy pathway, and formation of autophagy-related membranous structures at early steps of the exophagy pathway, it cannot be excluded that these processes could be related to the SMC disease state. Accordingly, studies investigating the functionality of autophagy, and indeed exophagic events in patient cells would be extremely valuable. In the future, we hope to gain a more complete understanding of the functional intertwining between membrane trafficking, autophagy and secretion, in conjunction with a clarification of the many roles of Rab33b, which will provide an opportunity to dissect Rab33b-mediated pathways and how they contribute to human disease.

## Figures and Tables

**Table 1:** Rab GTPase proteins, and their autophagic effectors/interactors that function in autophagosome biogenesis and maturation.

**Mammalian Rab GTPases That Function in the Autophagic Pathway Contributing to Autophagosome Biogenesis**			
**Rab Protein**	**Autophagy Effectors/Interactors**	**Function in Autophagy**	**References**
Rab1a Rab1b	Atg5, ULK1 Unknown	Translocation of ULK1 complex and mAtg9-containing vesicles at pre-autophagosomal membranes is Rab1a-dependent. Activity of Rab1b is required for autophagosome formation at ER exit sites, it may regulate the amount of PI(3)P in the omegasome through interaction with myotubularin-related protein 6.	[30,31,32,33,34,35]
Rab3b Rab3d	LC3 Atg16L	The GTPase-activating domain of RAB3GAP1/2 cooperates with Atg3 or Atg16L to sustain autophagosome biogenesis. Indirect evidence for involvement of Rab3 in autophagosome biogenesis.	[30,36]
Rab4a	Unknown	Formation of LC3-positive autophagic structures in response to overexpression of Rab4, following localisation to those structures upon blockade of mTORC1.	[37]
Rab5c Rab5	LC3, Atg10, PIK3C3	Rab5 acts as an activator of the Vps34/Beclin1-PIK3C3 complex and promotes Atg5-Atg12 conjugation, which in turn leads to elongation of pre-autophagosomal structures. Rab5 forms part of a signalling cascade that promotes initiation of autophagy independently of nutrient shortage and controls mTORC1 activation and localisation.	[30,38,39,40]
Rab9a	Unknown	Rab9a function is required for generation of autophagosomes from *trans*-Golgi-derived IMs in Atg5- and Atg7-independent autophagy.	[41]
Rab11	MLST8, TBC1D14, ULK1, Atg16L	Rab11 mediates incorporation of recycling endosomal membranes that contain ULK1 and mAtg9 to the IM and modulates autophagosome elongation upon amino acid starvation. This process is negatively regulated by the non-GAP effector TBC1D14. Rab11a-positive membranes provide a platform for autophagosome biogenesis by favouring the recruitment of the Atg16L complex.	[30,42,43]
Rab12	Unknown	Regulates trafficking and lysosomal degradation of the amino-acid transporter PAT4. Loss of Rab12 results in accumulation of PAT4 and increased mTORC1 activity, which thereby inhibits autophagy.	[44,45]
Rab13	Unknown	Mediates pterostilbene-induced autophagy in endothelial cells via functional interaction of GTP-active form with growth factor receptor-bound protein 2 (Grb2), which leads to mTOR inhibition.	[46]
Rab14	Unknown	Functions in earlier stages of autophagosome formation; its silencing causes a reduction in the size of autophagic vesicles, whereas overexpression leads to the opposite effect.	[47]
Rab26	Atg16L	The GTP-form of Rab26 selectively recruits Atg16L and Rab33b into large clusters of synaptic vesicles that represent pre-autophagosomal compartments.	[48]
Rab32	Unknown	Rab32 facilitates the formation of LC3-positive autophagic structures from the ER membrane during basal autophagy.	[49]
Rab33b	Atg5, Atg16L	Regulates conjugation of LC3 to PE through recruitment of the Atg12-Atg5-Atg16L complex.	[30,50]
Rab39a	Atg5, Atg14L, PIK3C3, Beclin, Vps34	Negatively regulates autophagy induced by LPS in macrophages through PI3K/Beclin-dependent mechanisms.	[30,51]
**Mammalian Rab GTPases That Function in the Autophagic Pathway Contributing to Autophagosome Maturation**			
**RAB Protein**	**Autophagy Effectors/Interactors**	**Function in Autophagy**	**References**
Rab2a/b	HOPS complex	Promotes autophagosome clearance via its localisation to autophagosomes. Mediates *trans*-SNARE complex formation and coordinates fusion of amphisomes through the HOPS complex with Rab7-marked structures.	[52]
Rab7	UVRAG, RILP, FYCO1, CLN3, Rubicon, PIK3C2A, UBE1DC1	Main regulator of trafficking of autophagosomes and their fusion to lysosomes via effector proteins; binding to LC3 and PI(3)P through FYCO1 regulates Rab7-dependent transport of autophagosomes through microtubule tracks; RILP mediates binding to dynactin-dynein1; the component of the Beclin 1 complex, UVRAG, activates Rab7 through the GEF activity of HOPS complex; Rubicon inhibits Rab7 activation by blocking UVRAG function.	[30,53,54,55,56,57]
Rab11	Hook	Regulation at the level of fusion between autophagosomes and multivesicular bodies. *Drosophila* Rab11 removes the microtubule binding protein Hook, a negative regulator of endosome maturation, allowing subsequent fusion events.	[58,59]
Rab14	Klp98A	Through its effector Klp98A (*Drosophila* orthologue of human KIF16B kinesin 3 family member), Rab14 controls the positioning of lysosomes and promotes autophagosome-lysosome function.	[47]
Rab21	UBE1DC1, VAMP7, VAMP8	Rab21 endosomal activity promotes VAMP8 endo-lysosomal trafficking to Rab7-positive late endosomes and SNARE-mediated autophagosome-lysosome fusion, which results enhanced in response to starvation.	[30,60]
Rab24	Drs, Rab7, RILP	Following induction of autophagy Rab24 localises in spots decorated with LC3, mediating the clearance of late autophagic compartments after their acquisition of degradative capacity and upon nutrient-rich conditions. Its interaction with drs tumour suppressor regulates fusion with lysosomes. Interacts with Rab7/RILP	[61,62,63,64]
Rab33b	UVRAG, CLN3	Regulates the fusion of autophagosomes with lysosomes. Regulation by its GAP protein (OATL1) is necessary to ensure autophagosome maturation.	[30,65]
